# Matrix-induced autologous chondrocyte implantation for a large chondral defect in a professional football player: a case report

**DOI:** 10.1186/1752-1947-6-173

**Published:** 2012-06-28

**Authors:** Tahsin Beyzadeoglu, Ayberk Onal, Alan Ivkovic

**Affiliations:** 1 Department of Orthopedics and Traumatology, Yeditepe University Hospital, Faculty of Medicine, Istanbul, Turkey; 2 Department of Orthopedic Surgery, University Hospital Sveti Duh, Zagreb, Croatia; 3 Department of Orthopedic Surgery, St Catherine’s Hospital, Zabok, Croatia

## Abstract

**Introduction:**

Matrix-assisted autologous chondrocyte implantation is a well-known procedure for the treatment of cartilage defects, which aims to establish a regenerative milieu and restore hyaline cartilage. However, much less is known about third-generation autologous chondrocyte implantation application in high-level athletes. We report on the two-year follow-up outcome after matrix-assisted autologous chondrocyte implantation to treat a large cartilage lesion of the lateral femoral condyle in a male Caucasian professional football player.

**Case presentation:**

A 27-year-old male Caucasian professional football player was previously treated for cartilage problems of his left knee with two failed microfracture procedures resulting in a 9 cm^2^ Outerbridge Grade 4 chondral lesion at his lateral femoral condyle. Preoperative Tegner-Lysholm and Brittberg-Peterson scores were 64 and 58, and by the second year they were 91 and 6. An evaluation with magnetic resonance imaging demonstrated filling of the defect with the signal intensity of the repair tissue resembling healthy cartilage. Second-look arthroscopy revealed robust, smooth cartilage covering his lateral femoral condyle. He returned to his former competitive level without restrictions or complaints one year after the procedure.

**Conclusions:**

This case illustrates that robust cartilage tissue can be obtained with a matrix-assisted autologous chondrocyte implantation procedure even after two failed microfracture procedures in a large (9 cm^2^) cartilage defect. To the best of our knowledge, this is the first case report on the application of the third-generation cell therapy treatment technique, matrix-assisted autologous chondrocyte implantation, in a professional football player.

## Introduction

Articular cartilage injuries commonly occur after blunt trauma and ligamentous knee injuries. These injuries result in the impaction of cartilage tissue, causing softening, flap tears, cracking or delamination in the young adult knee. Such defects do not heal and, with time, often lead to premature osteoarthritis, consequently decreasing quality of life for the patient and adding substantially to health care costs. The degree of morbidity depends on the location, size and grade of the lesion, and treatment options depend on mentioned factors as well as on patient age and demand. Traditional marrow-stimulating (microfracture) and cartilage restoration (mosaicplasty) techniques alleviate pain and restore function, but may not be satisfactory for high demand patients, such as professional athletes with large chondral lesions.

Autologous chondrocyte implantation (ACI) is a well-known procedure for the treatment of cartilage defects, which aims to establish a regenerative milieu and restore hyaline cartilage [1]. However, much less is known about third-generation ACI application in high-level athletes. We report on the two-year follow-up outcome after matrix-assisted autologous chondrocyte implantation (MACI; Genzyme, Copenhagen, Denmark) in the case of a large cartilage lesion of the lateral femoral condyle in a professional football player previously treated with two failed microfracture procedures.

## Case presentation

A 27-year-old male Caucasian professional football player had recurrent pain and swelling of his left knee. Seven years ago at another center, our patient underwent arthroscopic reconstruction of his left anterior cruciate ligament with a bone-patellar tendon autograft and partial lateral meniscectomy. His present complaints began one year after the initial surgery, and a large chondral lesion was detected on his lateral femoral condyle. On two occasions our patient had undergone microfracture procedures, but had been unable to return to the professional sport due to constant pain and swelling of his knee. On examination, our patient had full extension and 130° of flexion, and elicited pain on the lateral side of his knee after 60° of flexion. He had an effusion and loose bodies were palpated in the lateral recess. Anterior drawer, Lachman and McMurray tests were negative. Tegner-Lysholm and Brittberg-Peterson scores were 64 and 58, respectively. Neurologic and vascular examinations were normal.

**Figure 1:**
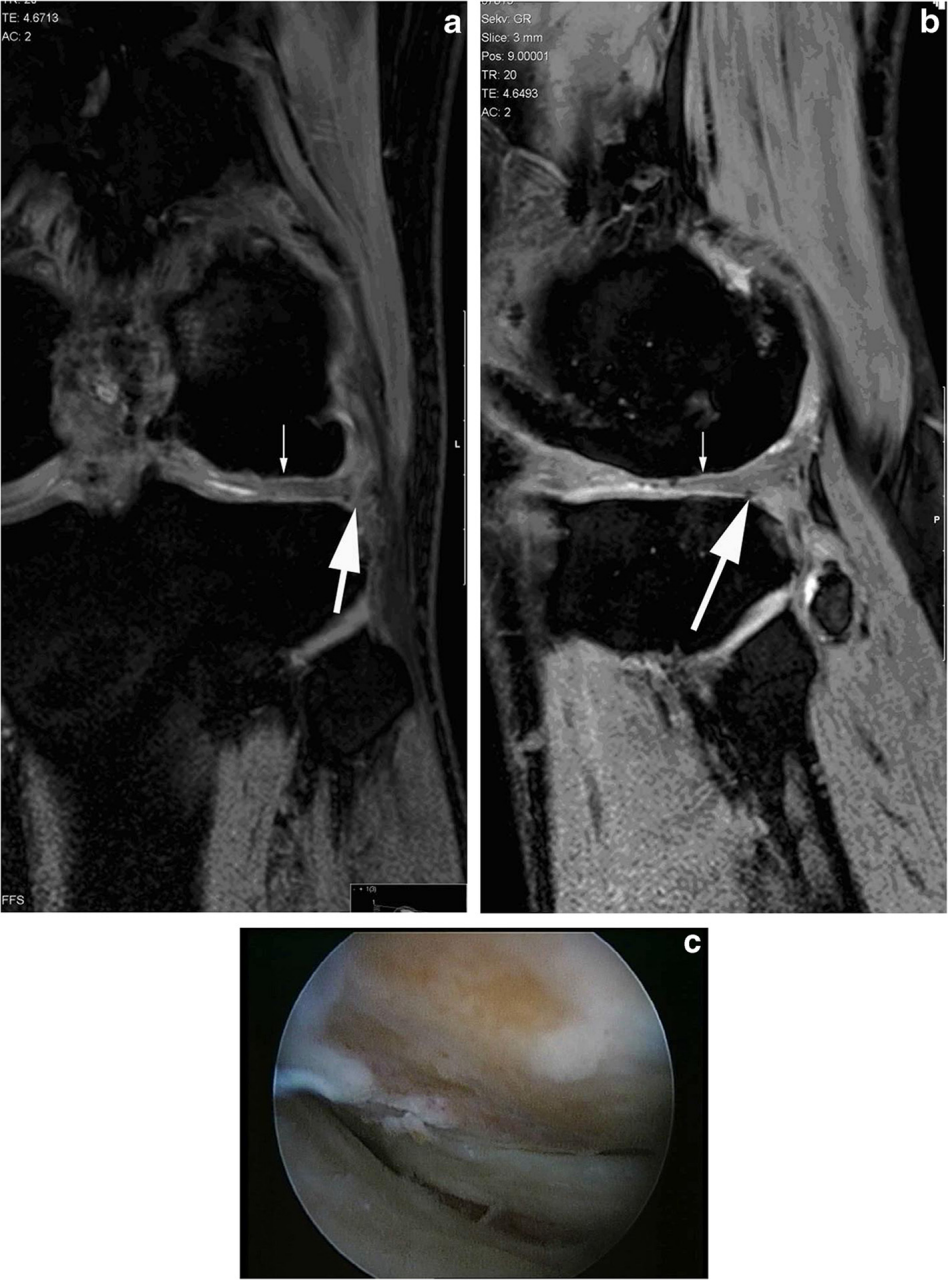
**Magnetic resonance imaging of the knee on presentation.** Anterior cruciate ligament reconstruction and two microfracture procedures had been performed on the left knee during the course of the last seven years. (**a**) Sagittal and (**b**) coronal three-dimensional gradient echo T1 (WATSf; repetition time 20, echo time 5.2, field of view 150, fractional anisotropy 15°, slice thickness 3 mm) left knee magnetic resonance images demonstrate a large cartilage defect on the lateral femoral condyle (arrow). Hypointense metallic artifacts due to the anterior cruciate ligament reconstruction and osteophyte formation (thick arrow) were also observed. (**c**) An Outerbridge grade 4, Noyes grade 3B 3 cm × 3 cm large chondral defect with a rough surface at the lateral condyle was diagnosed.

Magnetic resonance imaging (MRI) demonstrated a grade 4 large chondral lesion at the weight-bearing area of the lateral femoral condyle of his left knee (Figure 1a, b).

Informed consent was obtained from our patient and approval was obtained from the local ethics committee. An arthroscopic examination revealed a large chondral defect with a rough surface measuring 3 cm × 3 cm (Outerbridge grade 4, Noyes grade 3B) on the lateral femoral condyle (Figure 1c). Loose bodies were removed, and autologous cartilage biopsies were obtained from the superomedial edge of the femoral trochlea and sent to a commercial cell culturing facility.

Two months later, a MACI procedure was performed. With our patient under general anesthesia, the lesion was approached via a lateral 10 cm parapatellar incision. Osteophytes were excised, the cartilage defect was debrided to the subchondral bone with a 90° angled rougine, and the surface was smoothened with a low-speed burr. The defect size was measured as 3 cm × 3 cm (9 cm^2^) and MACI implant was resized over a template to match the defect size. A fibrin-containing tissue glue (Tisseel, Baxter Inc., Seoul, Republic of Korea) was used to fix the scaffold containing the cultured autologous chondrocyte within the defect. After five minutes in full extension, flexion and extension movements were performed to check for implant fixation. The wound was then closed layer by layer.

Our patient remained non-weight-bearing for six weeks postoperatively, began partial weight-bearing from the seventh postoperative week, and gradually progressed to full weight-bearing at 12 weeks. Range of motion exercises from 0° to 40° were started on the second day after the procedure, using continuous passive motion for four to six hours daily. After one week, the range of motion was increased by 5° per day. During this period, a quadriceps strengthening exercise and stretching of the hamstrings and calf were continued. Six months after the procedure our patient was allowed to jog and to initiate low velocity running. The physical therapy and fitness program were continued for nine months. Our patient was on the football field with full activity at the end of the first year.

At his last follow-up, two years after the index procedure, our patient had a full range of motion with no lateral knee pain or swelling. Tegner-Lysholm and Brittberg-Peterson scores were 91 and 6, respectively. He was playing in a professional football league and had a good performance within full season matches.

**Figure 2:**
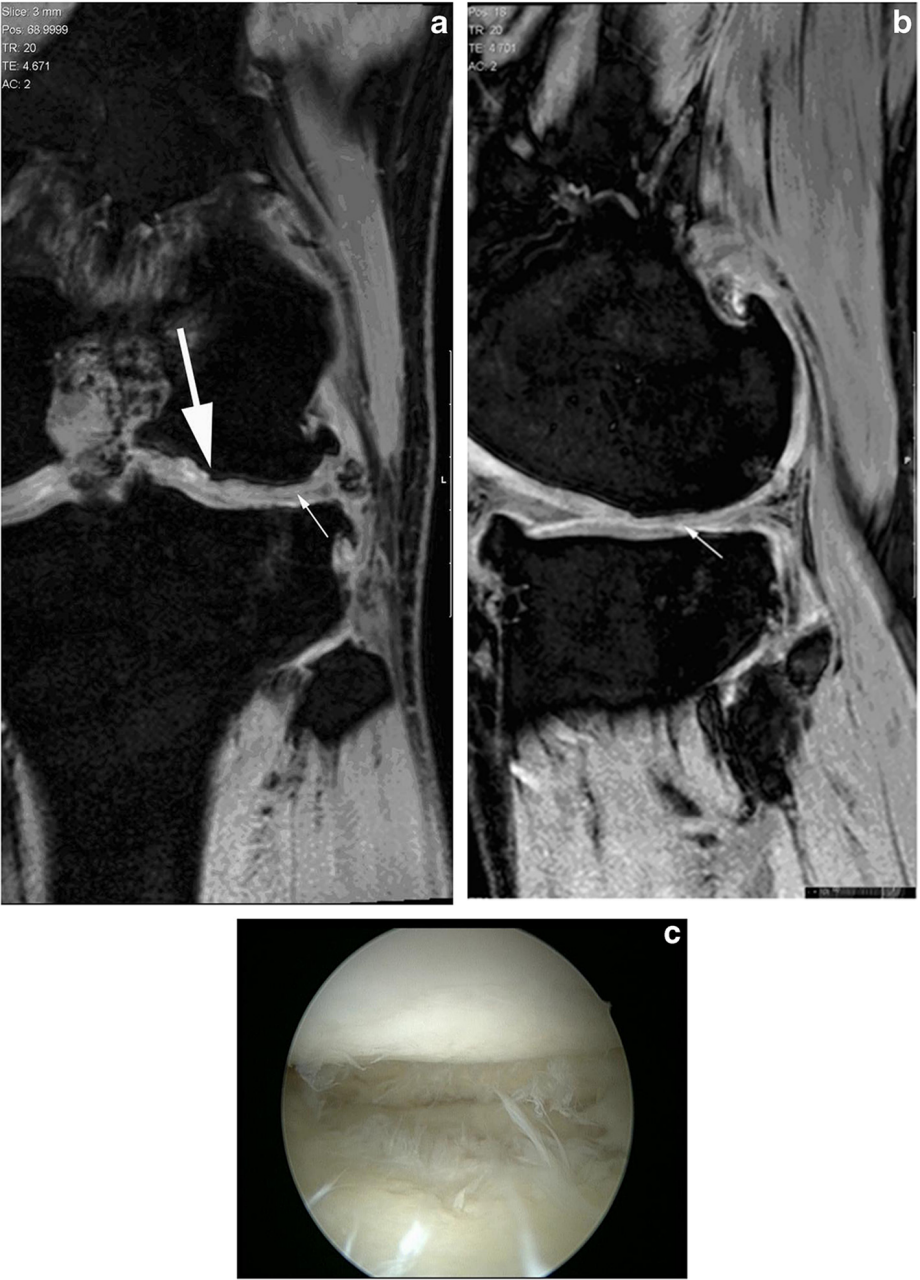
**Second year follow-up magnetic resonance imaging.** (**a**) Coronal and (**b**) sagittal three-dimensional gradient echo T1 (WATSf; repetition time 20, echo time 5.2, field of view 150, fractional anisotropy 15°, slice thickness 3 mm) left knee magnetic resonance images demonstrate the morphology of the cartilage implant. The graft is well integrated with a thickness that is similar to that of the adjacent cartilage; the graft surface is smooth (arrow). Mildly decreased signal intensity and artifacts are observed in the graft compared with the adjacent cartilage. Underneath, the large graft irregular bone contour (thick arrow) and osteophyte formation at bony margins is also noted. **(c)** Second-look arthroscopy at two years demonstrated complete filling of the defect and excellent integration of the newly formed cartilage with native tissue.

MRI was performed (Philips, Achieva 3 T, Amsterdam, Netherlands) every six months. An MRI evaluation two years after the MACI confirmed that our patient maintained an excellent repair of the defect under the high demands of professional soccer (Figure 2a, b).

Our patient had no complaints, but a second-look arthroscopy was performed to evaluate the outcomes of the MACI procedure at two years. Informed consent was taken. The cartilage defect was totally healed with the same margin to the normal cartilage and as robust as healthy cartilage (Figure 2c). Our patient did not give permission for a biopsy.

## Discussion

The treatment of cartilage defects in young athletes is still a challenging situation for the treating physician. Early recognition and diagnosis is important, and the most appropriate treatment depends on various factors such as size of the lesion, level of activity and age of the patient. Current treatment modalities include microfracture, transplantation of osteochondral grafts and ACI, each having its own benefits and shortcomings.

Microfracture and other bone marrow stimulation techniques involve penetration of the subchondral plate to recruit mesenchymal stem cells into the chondral defect. The formation of a stable clot that fills the lesion is of paramount importance to achieve a successful outcome. The technique is safe, easy and cheap, with excellent short-term results when used in small cartilage defects [2]. However, the resulting fibrocartilage repair tissue predominantly contains collagen type I, which cannot provide enough shear and tensile strength in comparison with native hyaline cartilage that contains 90% to 95% of type II collagen fibers. In a recent prospective, randomized study including 60 patients with a symptomatic, post-traumatic, single, isolated chondral defect, Basad *et al.* showed that MACI was significantly more effective over time (24 months versus baseline) than microfracture, according to the Lysholm (*P* = 0.005), Tegner (*P* = 0.04), International Cartilage Repair Society patient (*P* = 0.03) and International Cartilage Repair Society surgeon (*P* = 0.02) scores [3].

Minas and coworkers recently demonstrated a three-fold increase in the failure rate of ACI when performed after microfracture [4]. They suggested that the defect bed should be prepared with micro-burring to remove the stiffened subchondral bone and intralesional osteophytes, which should decrease the risk of failure over the long term. Failed microfracture procedures alter the smoothness of subchondral bone, thus transforming the defects from Noyes grade 3A to 3B. Our patient had two failed microfracture procedures before the MACI; therefore, we used a low-speed micro burr to carefully remove the calcified cartilage down to the subchondral bone plate to prepare the defect bed for construct implantation. The surgeon must be cautious about bleeding while micro-burring, as bleeding between the implant and subchondral bone can delaminate the implant.

Autologous osteochondral grafting (mosaicplasty) provides better functional results in comparison with marrow stimulation techniques [5]. However, donor site morbidity following the treatment of large defects may limit clinical usage of this method. Bentley *et al.* showed significant superiority of ACI over mosaicplasty for the repair of knee articular cartilage defects in a prospective, randomized, clinical trial with 60 patients [6]. Modified Cincinnati and Stanmore scores and objective clinical assessment showed that 88% of patients had excellent or good results after ACI compared with 69% after mosaicplasty. Arthroscopy at one year demonstrated excellent or good repairs in 82% of patients after ACI and in 34% of patients after mosaicplasty.

Although there are several long-term follow-up studies on ACI used in professional athletes, much less is known about the clinical outcome of the third-generation cell therapy treatment techniques such as MACI when used in the same population [7-9]. Peterson *et al.* reported that the average Lysholm score was improved from 60.3 preoperatively to 69.5 postoperatively, the Tegner from 7.2 to 8.2, the Brittberg-Peterson from 59.4 to 40.9, and the Knee injury and Osteoarthritis Outcome Score was on average 74.8 for pain, 3 for symptoms, 81 for activities of daily living, 41.5 for sports and 49.3 for quality of life after a mean 12.8 years follow-up (range: 10 to 20 years) of 224 patients [7]. Mithöfer *et al.* reported good to excellent results, with significant overall improvement of Tegner activity rating scale scores after articular cartilage repair in soccer players treated with ACI [8]. In their study, 33% of the players returned to soccer, including 83% of competitive-level players and 16% of recreational players. Of the returning players, 80% returned to the same skill level and 87% maintained their ability to play soccer at 52 ± 8 months postoperatively.

This case illustrates that robust cartilage tissue can be obtained with the MACI procedure even after two failed microfracture procedures in a 9 cm^2^ cartilage defect. The newly formed cartilage can provide shear and tensile strength sufficient for a full-capacity training regime in a top-level athlete, as full function was regained 12 months after the procedure in a professional football player.

## Conclusions

MACI can be a very good treatment option for professional athletes with massive cartilage lesions of the knee, even after failed marrow-stimulating techniques. Comparative advantages of MACI over ACI include shorter surgical time, less invasive fixation method (fibrin glue instead of transcartilaginous sutures) and more even distribution of cells within the three-dimensional scaffold. Clinical outcome and second-look arthroscopy at two-year follow-up were very encouraging, and our patient is still playing football in a professional league. To the best of our knowledge, this is the first case report on the application of the third-generation cell therapy treatment technique, MACI, in a professional football player, and further long-term follow-up studies with large series of patients are necessary to fully assess the true potential of this procedure.

## Consent

Written informed consent was obtained from our patient for publication of this case report and accompanying images. A copy of the written consent is available for review by the Editor-in-Chief of this journal.

## Competing interests

The authors declare that they have no competing interests.

## Authors’ contributions

TB performed the surgery of the patient with the assistance of AO. AO analyzed and interpreted the patient with AI and prepared the manuscript. All authors read and approved the final manuscript.
